# Perceptions, knowledge and beliefs of women regarding second-hand smoke exposure: a qualitative study to identify approaches for change

**DOI:** 10.3389/fpubh.2026.1716775

**Published:** 2026-02-05

**Authors:** Hatice Bulut, Gamze Nalbant, Zeinab M. Hassanein, Turki S. Alqurashi

**Affiliations:** 1 Department of Nursing, Division of Obstetrics and Gynecologic Nursing, Faculty of Health Sciences, Suleyman Demirel University, Isparta, Türkiye; 2 Department of Nursing, Division of Public Health Nursing, Faculty of Health Sciences, Suleyman Demirel University, Isparta, Türkiye; 3 Public Health and Community Medicine Department, Faculty of Medicine, Assiut University, Assiut, Egypt; 4 Department of Social Work, Al-Lith University College, Umm Al-Qura University, Mecca, Saudi Arabia

**Keywords:** second-hand smoke, women’s health, children’s health, qualitative study, thematic analysis, nursing

## Abstract

**Purpose:**

This study aims to reveal women’s perceptions, knowledge levels, beliefs, and exposure experiences regarding second-hand smoke (SHS) and to examine their protective strategies accordingly.

**Method:**

The research was conducted in Türkiye between March and August 2025. Ten women, mostly university graduates and aged between 30 and 42 (mean age = 34.7), participated in the study. Data were collected using a semi-structured interview form, audio recordings were analysed, and thematic analysis was used.

**Findings:**

Although none of the participants smoked, they were found to be regularly exposed to SHS through their spouses, family members, or public spaces. The analysis revealed five main themes: (1) Awareness of and Perceived Harm from SHS, (2) Gender, Patriarchal Structure, and Silence, (3) The Permeability of Boundaries: “Smoke is Everywhere,” (4) Resistance Strategies: Creating Smoke-Free Sanctuaries, and (5) The System’s Response: From the Visible to the Deeper Issues. Findings indicate that women demonstrate active resistance by making their homes smoke-free and raising awareness among their children; however, patriarchal relations, cultural norms, and lack of counselling in the healthcare system limit these efforts.

**Conclusion:**

SHS exposure continues to be a significant public health problem for women and children. For women’s individual awareness and resistance efforts to be effective, the role of counselling by health professionals must be strengthened, public awareness campaigns must be increased, and more comprehensive policies must be developed to include domestic and private spaces.

## 1 Introduction

Exposure to second-hand smoke (SHS) continues to be a pressing global health challenge (1). Among those most vulnerable are pregnant women and children, since SHS during pregnancy and early childhood can cause serious harm to both foetal development and child health (2). In Middle Eastern countries, cultural restrictions on women’s smoking contribute to higher smoking prevalence among men, leaving non-smoking women and children more likely to be exposed (3). During pregnancy, SHS is considered a preventable determinant of developmental complications and is associated with adverse perinatal and postnatal outcomes that often persist into infancy and beyond. The harmful consequences can even occur in utero, as the placenta does not block SHS exposure (4).

Exposure to SHS during childhood has been strongly linked with both morbidity and mortality, as well as substantial economic costs arising from healthcare use, behavioural difficulties, and an increased likelihood of smoking uptake in later life (5). Research consistently shows that infants and children exposed to SHS are more prone to sudden infant death syndrome (SIDS), respiratory infections, asthma, middle ear infections, invasive meningococcal disease, behavioural problems, psychological issues, and eventual smoking initiation (5–8). Many of these health conditions may require hospitalisation, and prolonged illness can disrupt growth, education, and behaviour. The broad negative consequences of SHS are entirely preventable; therefore, protecting pregnant women and children from SHS exposure must be prioritised in public health (3). Healthcare professionals are encouraged to ask expectant mothers and caregivers about SHS exposure, raise awareness about its risks, and provide guidance on prevention strategies.

Comprehensive smoke-free laws are widely recognised as an effective public health measure, with meta-analyses and Cochrane reviews from multiple countries showing significant reductions in cardiovascular and respiratory hospitalisations (9, 10). Similarly, significant improvements in serious perinatal outcomes, such as stillbirth and neonatal deaths, were observed following the implementation of a national law in the United Kingdom (11). However, this proven success in public spaces has increasingly shifted the focus of combating SHS to private spaces and at home. For example, a study from the United Kingdom showed that despite public bans, indoor exposure remains insufficient, and children of parents who smoke enjoy only partial protection (12). This demonstrates that legal regulations alone are not sufficient, and that complex factors such as cultural norms and family dynamics come into play in the home environment, where SHS exposure is most intense. Therefore, a thorough understanding of the dynamics in this area is crucial for effectively protecting vulnerable groups such as women and children.

A systematic review of qualitative studies examining parental perspectives on preventing second-hand smoke exposure in Middle Eastern contexts identified several themes (13). Parents were generally aware of SHS harms, yet discussions about SHS risks were rarely included in antenatal care. Smoking was described as a culturally embedded behaviour, with traditional norms limiting parents’ ability to establish smoke-free homes. Although some families attempted household restrictions such as designated smoking areas, they often felt uncertain about the necessity and effectiveness of these strategies (13).

Data from the Global Adult Tobacco Survey conducted in 2016 show that tobacco use rates in Türkiye are 44.1% in men and 19.2% in women (14). Tobacco use continues to impose a substantial health burden in Türkiye. Global Burden of Disease 2019 Türkiye Report results showed that tobacco-attributable disability-adjusted life years (DALYs) per 100,000 population increased from 3,283 in 2002 to 3,407 in 2019 (15). It is reported that exposure to SHS smoke, especially at home, is a significant problem in Türkiye with approximately 39% of adults and 56% of children exposed within their households (16). This creates a critical gap for Turkish women and children, who are disproportionately affected by SHS but often lack the power to negotiate for smoke-free environments. Thus, the current study was performed to explore Turkish women’s perceptions, knowledge, beliefs, exposure status and the obstacles they face in preventing exposure to passive smoking; in light of these findings, policy makers in ministry of health can support women’s and children health in particular and to develop effective solution strategies to prevent potential negative effects.

## 2 Methods

A qualitative research design was selected for its strength in capturing the complexity, processes, and underlying meanings of individual actions. This phenomenological qualitative study was conducted in Türkiye from March to August 2025. Reporting of this study was guided by the Consolidated Criteria for Reporting Qualitative Research (COREQ-32) guideline (17). An ethics approval was obtained from the ethics committee of the Suleyman Demirel University (Ethics no: Approval Date: 25.03.2025, Decision No: 48). At the start of each interview, participants received a verbal explanation of the study purpose, procedures, potential risks, and their rights, including voluntary participation and the option to withdraw at any time. Verbal informed consent was obtained and audio-recorded. All audio files and transcripts were stored on a password-protected, encrypted computer accessible only to the research team. Identifying information was removed during transcription, and participants were assigned numerical codes.

### 2.1 Interview guide

A semi-structured interview guide was initially developed from the literature and subsequently through discussions within the research team, and covered knowledge of hazards, attitudes towards smoking and SHS, experiences of exposure to smoking at home, work or outside, and barriers and facilitators to the prevention of SHS. See Supplementary file 1.

### 2.2 Study participants, sample size and recruitment

Participants were pregnant women or mothers of children under 7 years of age (i.e., mothers of preschool-aged children), who were non-smokers, over 18 years old, and were able to speak and understand Turkish. Thirteen participants were approached; however, three did not follow up to schedule an interview, citing a lack of time. The remaining 10 participants were recruited via Facebook and Instagram advertisements, as well as WhatsApp messages, using a snowball sampling approach. No incentives were provided. We intended to interview 13 women, aiming to achieve saturation of themes. However, the saturation of themes was achieved at the 8th interview, and we continued to interview two more women to ensure that any important theme was not missed.

Verbal consent was taken at the beginning of the interview. Interviews were conducted by two researchers (HB/GN), trained in qualitative research methods, in Turkish using the interview guide. Interviews were conducted in person or through video conferencing. With the consent of the participants, the interviews were digitally audio recorded. Participants were informed that transcripts would be anonymised and treated confidentially and that they were free to withdraw at any point during the interview if they so wished. Interviews lasted between 40 min and 1 h and 10 min.

Field notes were collected alongside the interviews. One researcher (HB) transcribed the recordings verbatim, and the accuracy of the transcripts was checked by the second researcher (HB/GN). All identifying details were removed, and transcripts were assigned numerical codes.

### 2.3 Data management and analysis

Transcripts were analysed in a word document. Data were analysed using latent thematic approach and inductively so that the researchers could determine the important concepts from the participants’ perspectives (18). Interviews were read several times by one researcher (HB/GN) and then the transcripts were coded line by line. A second researcher then reviewed the coding to ensure accuracy and consistency. The preliminary codes were discussed between the researchers and if any discrepancies were found, these were discussed with other researchers (ZH and TA). Then, codes were organised into overarching categories, which formed themes. Codes and themes were subsequently discussed among all the researchers resulting in a thematic codebook. All researchers examined the themes to confirm they were clearly defined and non-overlapping. Themes were then reviewed in the context of the entire data set to ensure they accurately represented it. The analysis followed an iterative approach, with codes being refined continuously as new insights emerged. Each theme was presented with illustrative quotations selected for their ability to best capture the essence of that theme. Participants were not asked to provide feedback on the results.

The data were analysed by two independent researchers using the thematic analysis of Braun and Clarke (18). This analysis was preferred over other types of analysis because it is used to illustrate meanings, experiences and reality of participants. The Braun and Clarke’s phases of qualitative data analysis are: familiarization, generating initial codes, searching for themes, reviewing themes, defining and naming themes and reporting. The researcher (HB) followed these steps. Firstly, the researcher read the transcripts more than one time to be familiar with the data. Transcripts were read again until themes and sub-themes emerged, resulting in an analytical framework of main themes and sub-themes. The researcher indexed the data using the identified themes and sub-themes. Each interview was analysed separately; similar codes generated from each interview were grouped together to generate findings. Quotations were used to serve as supporting evidences for the themes generated. The generated themes and sub-themes were discussed between independent researchers (ZH and GN), which resulted in clarification of the final thematic framework.

### 2.4 Reflectivity

Interviews were conducted by two female researchers with doctoral training and experience in qualitative research. Both researchers were mothers, a factor that may have contributed to empathy and rapport with participants. The researchers’ positionality as mothers may have shaped both the interview dynamics and the interpretation of data. However, to address this, the researchers engaged in reflexive practices throughout the study, including keeping field notes and discussing emerging interpretations with other two researchers to minimise the influence of personal beliefs and experiences on the analysis. To reduce the influence of interviewer characteristics, both researchers followed a reflexive protocol that included using neutral questioning techniques, documenting assumptions before and after interviews, and discussing potential biases with the wider research team.

## 3 Findings

This study’s sample included 10 married women aged 30 to 42 (mean age: 34.7), who were predominantly employed (*n* = 10) and educated at the university level (eight with bachelor’s degrees, two with master’s). The participants’ financial situations were mixed, as five reported a balanced budget, three earned more than their expenses, and two earned less; concurrently, nine of the women had children and one was pregnant. A key health-related finding was that despite none of the participants being smokers, all experienced exposure to second-hand smoke from various sources, including spouses (*n* = 5), other family members, and public environments. Additionally, three participants reported taking regular medication for chronic conditions such as Hashimoto’s, asthma, and allergies (see Table 1).

**Table 1:** Demographic characteristics of participants (*N* = 10).

Participant	Age	Education	Employment status	Children	Pregnancy	Spouse smoke	Income status	Chronic illness	Medication use	Second-hand smoke exposure
1	34	Bachelor’s	Employed	2	No	No	Equals expenses	Yes	Yes	Yes
2	37	Bachelor’s	Employed	1	No	Yes	Less than expenses	No	No	Yes
3	37	Master’s	Employed	2	No	Yes	Less than expenses	No	No	Yes
4	42	Master’s	Employed	2	No	Yes	Less than expenses	No	No	Yes
5	30	Bachelor’s	Unemployed	2	No	No	Equals expenses	No	No	Yes
6	40	Bachelor’s	Employed	2	No	No	Equals expenses	Yes	Yes	Yes
7	33	Bachelor’s	Employed	1	No	Yes	More than expenses	Yes	Yes	Yes
8	32	Master’s	Employed	2	No	No	More than expenses	No	Yes	Yes
9	31	Bachelor’s	Unemployed	0	Yes	Yes	More than expenses	No	No	Yes
10	31	Bachelor’s	Employed	1	No	No	Equals expenses	Yes	Yes	Yes

As a result of the thematic analysis, five main themes and 13 sub-themes were identified. The findings reveal the social, cultural, and structural dimensions of SHS, going beyond the individual life experiences of the women. Before presenting the five themes, it is important to outline the conceptual structure that connects them. Women’s experiences of SHS formed an interconnected pattern shaped by multiple levels. Individual factors such as basic knowledge and sensory discomfort influenced how women perceived risk. These perceptions were closely linked to interpersonal dynamics, including gender roles, family hierarchy, and maternal responsibilities. Environmental exposures in public, social, and private spaces further intensified vulnerability. In response to these combined influences, women developed various resistance strategies to protect themselves and their children. Finally, these lived experiences revealed broader system-level gaps in healthcare practices, educational efforts, and tobacco control policies. Together, these interconnected levels shaped the overall framework within which the five themes emerged.

### 3.1 Awareness of and perceived harm from SHS

Women had a general awareness of SHS, shaped more by personal experiences and concerns—especially for their children—than by detailed information. Their sensitivity to their own and their children’s health strongly reinforced their perception of harm. This main theme was analysed under two interrelated sub-themes: General Level of Awareness and Heightened Sensitivity Regarding Women’s and Children’s Health.

#### 3.1.1 Levels of awareness: “we know it’s harmful, but we do not know the details”

All participants interviewed had a general awareness that SHS was harmful. However, it was observed that they lacked detailed knowledge about its physiological effects, mechanisms, or long-term consequences. The perception of harm was evidently based on a general consensus rather than scientific details, with personal experiences and social observations compensating for this knowledge gap. It was noted that this situation transformed into a heightened concern, particularly with motherhood. This shows that women’s SHS risk perception is shaped more by social learning and maternal roles than by biomedical information.

"I think a child could be more affected because their lungs aren’t developed. But honestly, I don’t know that for a fact, scientifically." (Participant 1)

"Of course SHS is harmful. But I don’t know the full details. It damages the lungs, but how? I don’t know." (Participant 5)

Participants believed that SHS posed serious risks not only for children and infants but also for their own health. They frequently expressed that it could have negative effects specifically on women’s health, for instance, in areas like fertility, blood circulation, and hormonal balance. However, they also stated that they lacked clear information about how or in what manner these effects occurred. Furthermore, their statements indicated that this awareness was based not only on abstract knowledge but also on direct and unpleasant physical experiences, such as the smell of cigarette smoke.

"I think it affects women's health. But I don’t know exactly what it causes. I mean, I suspect it could be related to fertility." (Participant 4)

"Cigarettes smell really bad to a non-smoker. I was never exposed to it this much until I got married." (Participant 9)

Participants’ perception of SHS’s harmfulness was largely based on their own experiences and observations. Some noted this harm not only in terms of health but also in terms of its negative effects on appearance. For example, Participant 7 expressed concerns about both aesthetics and women’s health by stating, “It changes the colour of nails and reduces fertility.” This reflects a connection women make between appearance, femininity, and health risks.

#### 3.1.2 Heightened dangers regarding women’s and children’s health

Pregnancy and motherhood were among the most powerful factors that heightened the women’s sensitivity to SHS. Participants stated that they perceived it as a threat not only to their own health but also to the health of their unborn babies and young children. The belief that the air a mother breathes during pregnancy passes directly to the baby creates a strong motivation for protection. This reflects a maternal-protection framework where risk perception becomes stronger during pregnancy.

"When I was pregnant, I said to myself, whatever I breathe, the baby breathes too." (Participant 1)

Concerns were frequently voiced, such as the possibility that cigarette smoke could pass to the baby via the placenta, increase the risk of miscarriage, and negatively impact the mother’s psychological state:

"Cigarette smoke might be passing to the baby through the placenta." (Participant 6)

"…because there's this idea that whatever we eat goes to the baby, I think that whatever we breathe from the air also goes to the baby." (Participant 8)

Some participants reported observing health problems in the children of women in their social circle who had smoked during pregnancy. It was understood that these observations, while not based on direct scientific evidence, were powerful narratives in which participants constructed their own cause-and-effect relationships based on negative examples from their environment.

"The children who were born were more hyperactive, had a stutter… I had friends who smoked when they were pregnant. For instance, her child wears glasses now. The child has a stutter." (Participant 2)

"A friend of my mother's was like that… She had smoked. The child's fingers were missing… I think it was because of the cigarettes. Because she used to smoke a lot." (Participant 2)

Underlying this sensitivity were not only physiological anxieties but also the role of motherhood and parental values. The women expressed feeling intense discomfort with situations that exposed their children to smoke and stated that they made a conscious effort, both before and after birth, to ensure that smoking did not become a negative role model for their children.

"I can't stand it when I see someone smoking next to their own child." (Participant 4)

Some participants emphasized that the harm from smoking posed a risk not only through “exposure” but also in terms of how children perceive smoking. They noted specifically that the scent of flavoured electronic cigarettes could be deceptive to children and prevent them from recognizing the danger.

"Someone is smoking a vanilla-flavoured e-cigarette. The smell is pleasant. Kids wouldn't understand the harm." (Participant 8)

In conclusion, this theme revealed the core elements underlying the women’s attitudes toward SHS. These elements included a general knowledge of health risks, as well as the protective instinct heightened by motherhood, negative examples drawn from their environment, and the personal discomfort caused by exposure to cigarette smoke.

### 3.2 Gender, patriarchal structure, and silence

Participants’ attitudes toward SHS were found to be shaped not only by health concerns but also by intra-familial hierarchy, gender roles, and cultural norms. Two primary sub-themes were identified within this theme: Limited Intervention Due to Hierarchy and ‘Respect,’ and Silent Acceptance: The Normalization Tendency Among Women.

#### 3.2.1 Limited intervention due to hierarchy and ‘respect’

Many participants stated that they attempted to intervene in the smoking behaviour of male family members. There were examples where this intervention could be directed specifically at the father figure. However, the prevailing tendency was that these interventions remained limited, and the concept of ‘respect’ prevented direct objection. The participants’ narratives revealed that this hesitancy toward authority figures such as fathers, husbands, and fathers-in-law pushed women toward passive or indirect methods of intervention. It was expressed that underlying this behaviour was the cultural norm of ‘respect for elders’. This shows how patriarchal authority restricts women’s agency and limits their ability to protect themselves and their children from SHS.

"I can say something to my father, but most people can't. After all, he is an elder, and telling him to go out on the balcony in the cold seems rude to people, but I say it." (Participant 5)

It was seen that directly opposing the smoking behaviour of older individuals or a spouse’s family was perceived as “disrespectful” by some women. Therefore, to avoid offending the other party and to preserve ‘respect,’ these women limited their interventions when expressing their discomfort.

"In order not to offend, and also so it wouldn’t be disrespectful." (Participant 1)

"If it were my own mother, I could say something, but when it's my mother-in-law, my husband's family, there is a hesitation. It feels like you can't say it out of respect." (Participant 8)

#### 3.2.2 Silent acceptance: the normalization tendency among women

It was observed that women who grew up with male figures who smoked from their childhood generally regarded the presence of smoking in the home as a “normal situation.” Participants’ statements suggested that within cultural codes, men’s smoking is considered more natural, leading to the habit being perceived as an act identified with men.

"When I was little, it was always the men who smoked. The women wouldn't say anything. That's what we saw. We actually normalized it." (Participant 5)

It was stated that this cultural acceptance made it difficult for some women to intervene directly in smoking behaviour, and at times, even the husband’s family did not play a supportive role in the quitting process.

"Even his mother couldn't convince my husband to quit. I don't think his social circle has an effect anymore." (Participant 9)

Some participants, however, interpreted this situation not just as a cultural habit but also as a reflection of male dominance in daily life. In this context, smoking was seen not merely as an addiction but also as a practice that reinforces male authority within the family.

"I think that's what being patriarchal is… The woman has no say… The man is above everything, and so on." (Participant 4)

"My father would sit wherever he smoked, and we were in the same room. My mother couldn't say anything either. The woman had no authority there." (Participant 5)

On the other hand, in contrast to this general tendency of acceptance, it was also observed that some women took on a more active role to prevent their children from learning and adopting smoking, supervising their husbands and developing concrete strategies to prevent smoking inside the home. This indicates emerging resistance, where motherhood motivates women to challenge established norms.

"My husband smokes in secret so the children don't learn, which is why I warn him immediately if the smell gets on his clothes, etc." (Participant 4)

### 3.3 The permeability of boundaries: “smoke is everywhere”

One of the most dominant themes in the participants’ narratives was the perception of the inevitability of SHS exposure and its pervasiveness in all areas of life. Smoke was seen as a threat that transcended physical and social boundaries, infiltrating private and public spaces. It became clear that exposure was not limited to sharing the same physical space, as smoke from clothing, neighbouring balconies, and ventilation shafts also emerged as significant sources. This situation demonstrated how easily the boundaries women drew to protect themselves and their families could be breached and that they lived in a constant state of vigilance. This main theme was examined under three sub-headings based on the spaces smoke infiltrated: public areas, social venues, and private living spaces.

#### 3.3.1 Invisible exposure in public spaces

Participants stated that they were exposed to SHS even in open areas, noting that this frequently occurred in children’s parks, cafes, seaside and walking areas. It was seen that being exposed to others’ smoke even during ordinary actions like walking down the street was perceived as a violation of personal space. Smoking even in children’s play areas was interpreted by participants as a lack of social responsibility and an instance of individual pleasure taking precedence over collective health.

"You're sitting at a cafe, you've chosen a non-smoking table, but the smoke from another table blows over to you with the wind." (Participant 4)

"It happens in children's parks. I warn them every time. It bothers me a lot." (Participant 4)

"They smoke while walking on the street, there is no place to escape because they smoke while walking." (Participant 7)

#### 3.3.2 The negotiation of social space

It was understood that social venues like cafes, restaurants, and workplaces were a constant area of negotiation for the participants. It was expressed that having the best and most spacious areas of these venues reserved for smokers created a sense of injustice and exclusion among non-smoking participants. It was seen that break times at the workplace created a dilemma, requiring exposure to smoke in order not to be isolated from their social circle.

"Actually, the best part of cafes belongs to the people who smoke. And that really annoys me too. The biggest, most spacious, most comfortable spots are used by people who smoke. I have to stay inside in an enclosed area. Why?" (Participant 2)

"'I smoke, so let's sit in the smoking section.' For example, this really annoys me… It means they don't respect my preferences." (Participant 4)

"… Whenever my colleagues who smoke go to a cafe for coffee, I sit with them. That means we sit in the garden section, so we are exposed." (Participant 3)

#### 3.3.3 The permeability of private space

Participants emphasized that even their homes, which they regarded as the safest place, could not provide complete protection from SHS, as smoke from neighbours often seeped into their living areas. This intrusion—through walls, windows, drains, and ventilation—was perceived as a violation of privacy and a disruption of the sense of sanctuary associated with the home. SHS creates a sense of boundary loss, reducing women’s control even within their own homes.

"The smell of cigarettes came from the bathroom drain. The neighbour was clearly smoking while taking a bath." (Participant 6)

"The cigarette my neighbour smokes in their kitchen reaches all the way to our bedroom. We can't even open the window anymore." (Participant 5)

Several participants described that smoke entered their homes from balconies or toilets, making it difficult to avoid exposure. Others pointed out that even when no one smoked indoors, the odour was carried inside on clothes after smoking outside, spreading throughout the house. This aligns with the concept of “thirdhand smoke,” where smoke residues cling to surfaces and fabrics, prolonging exposure within the home environment. This highlights the persistent and layered nature of SHS, extending beyond direct exposure.

### 3.4 Resistance strategies: creating smoke-free sanctuaries

It was seen that participants positioned themselves not merely as passive victims exposed to SHS, but as active agents who developed strategies against this situation and took control of their living spaces. It was understood that these resistance strategies ranged from setting individual boundaries to engaging in social confrontations and efforts to raise awareness in future generations. This main theme was examined around three different areas of strategy: protecting the home as a fortress, charting new routes in the outside world, and educating the next generation.

#### 3.4.1 The home as a fortress

It was understood that the participants’ most fundamental resistance strategy was to turn their own homes into a smoke-free fortress with a “zero-tolerance” policy. Despite the social pressures and hierarchy mentioned in previous themes, participants defined their homes as spaces where they had absolute control. The home functioned as a “fortress” where women could break the cycle of “staying silent” and impose their own rules without negotiation. This situation showed that they prioritized their family’s health over cultural norms such as hospitality.

"No one smokes inside the house. It's smoked outside. But inevitably, because it clings to clothes, you can still be exposed." (Participant 2)

"After smoking, I want him to brush his teeth and change his clothes. I don't want any harm to come to the child." (Participant 4)

Other participants described directing guests outside to smoke, or even closing windows to prevent smoke from balconies, underscoring the strict boundaries they established to protect the home environment.

#### 3.4.2 Charting a course in the outside world

It was seen that in environments outside the home, various strategies were adopted to manage exposure, ranging from passive avoidance to active confrontation. It was understood that participants consciously avoided risky environments or made venue choices that would reduce exposure without damaging social relationships. Women strategically balance social harmony with personal and maternal protection. However, it was noted that they exhibited more confrontational attitudes, risking social conflict, especially when their children’s health was at stake:

"…For example, we go out. In the winter with these three friends of mine, two of them smoke, and they smoke a lot… I want to sit in a warm place. After this happened a few times… I started trying to arrange the venue accordingly. You know, without being obvious. 'Oh, let's not go there, let's go here.' I don't actually tell them why I don't want to go there. Because then it becomes, 'you're the one who doesn't smoke.'" (Participant 2)

"…when I encounter it outside, I immediately try to move away somehow. To avoid the smell, to avoid being exposed." (Participant 9)

"It happens in children's parks. I warn them every time… I know I'll get a reaction, but I still warn them." (Participant 6)

#### 3.4.3 Educating the next generation

The participants’ resistance strategies also included long-term efforts aimed at creating a more conscious attitude in the next generation by educating their children about the harms of smoking. Motherhood motivates long-term resistance aimed at reshaping social norms for future generations. With the responsibility of motherhood, they were concerned with being role models, supervising both themselves and their husbands to ensure that their children did not normalize or internalize smoking. In this context, they believed that in addition to explaining the harms of smoking, keeping the act of smoking out of their children’s sight was also an important strategy.

"…I explained it in front of his paternal grandmother. And she adds things like, 'Look, I'm trying to quit, it's not a good thing at all'… I enable my child to establish cause-and-effect relationships." (Participant 8)

"The children don't even know about smoking. My husband smokes in secret, and I warn him immediately if the smell clings to his clothes." (Participant 4)

### 3.5 The system’s response: from the visible to the deeper issues

Participants expressed that existing tobacco control policies, healthcare system protocols, and educational strategies fail to adequately address the individual and social impacts of SHS. This theme included systemic gaps ranging from individual experience to the public sphere, from education to legislation, and participant suggestions on how these gaps could be transformed. The women’s narratives brought with them not only a demand for protection but also deeper demands such as for visibility, empowerment, and systemic ownership. This main theme was examined under three primary sub-headings: “The Clinical Blind Spot and Counselling Silence,” “Beyond the ‘It’s Harmful’ Discourse – Depth in Education and State Responsibility, “and “Empowerment of the Non-Smoker and Spatial Justice.”

#### 3.5.1 The clinical blind spot and counselling silence

It was noted that information and counselling regarding SHS are often overlooked within the healthcare system. Participants emphasized that even during sensitive periods like pregnancy, healthcare professionals did not proactively ask questions or offer guidance on this topic. This reflects a missed opportunity for preventive counselling in maternal and primary care. However, some rare examples also revealed how much of a positive impact effective counselling could have.

"When I was pregnant, the doctor only asked, 'Do you smoke?' He never asked if my husband smokes or if anyone smokes around me." (Participant 2)

"The midwife told me that my husband shouldn't come near me for 30 minutes and that he should change his clothes. That was the first time I had ever heard such things." (Participant 9)

#### 3.5.2 Beyond the “it’s harmful” discourse – depth in education and state responsibility

Participants stated that superficial messages like “smoking is harmful” are no longer effective; instead, they argued for the need for emotional, experience-based, and interactive education. It was thought that more creative and participatory methods, such as support groups where individuals with similar experiences come together or community-based events, could be effective. They also emphasized that education should not be limited to the individual and that institutional actors like the Ministry of National Education and the Ministry of Health should take on a more consistent and comprehensive responsibility. It was highlighted that education organized for non-smokers was also important for turning them into conscious individuals who could influence smokers in their families. Furthermore, it was emphasized that integrating education with different approaches such as art therapy, yoga, or being in nature could encourage participation.

"If they met someone who smoked and got, they would be more affected. Just explaining it isn't enough." (Participant 9)

"Education is provided, but only those who want to attend do. If it were mandatory and included a test, maybe people would pay more attention." (Participant 5)

"Let them provide education in schools, so children grow up learning." (Participant 1)

"Maybe making the education a bit more fun, like by taking walks in beautiful places … Yoga is very popular in Türkiye, so 'let's do yoga in this park, and at the same time, we will give you a short psycho-education session.'" (Participant 8)

Some women stated that children can learn from an early age through cartoons. She noted that these videos, watched together by families, can be effective in raising awareness about children’s health and SHS exposure. She also suggested that parents’ smoking habits could be noted in parent-teacher conferences or observation forms filled out at the beginning of kindergartens.

“For example, we, as women, often watch cartoons with our children a lot… I think cartoons are very effective in terms of fostering family engagement… This issue could be brought up in parent-teacher conferences in kindergartens… This child's parents smoke. This child is exposed to SHS.” (Participant 10)

Women want education that is participatory, practical, and supported by systemic structures rather than individual responsibility alone.

#### 3.5.3 Empowerment of the non-smoker and spatial justice

Participants expressed that non-smokers should be seen not just as individuals who need protection, but as active agents in the process of change. However, it was understood that for this role to be realized, clear legal regulations, spatial justice, and systemic support are necessary. At this point, it was emphasized that the separation of smoking and non-smoking areas should not remain merely symbolic but must be organized in a way that is genuinely protective.

"There's a non-smoking area, but what's the point when the next table over is smoking? Separate areas should be created, but with a proper separation." (Participant 4)

"…In America, they couldn't even smoke in the garden section. There was a yellow line. They could only smoke beyond that line." (Participant 3)

Participants emphasized that individual awareness alone is not sufficient to prevent SHS; stricter enforcement and deterrent penalties are necessary. They stated that police patrol parks and areas where children are present, and that those who violate the rules should be fined. They also stated that current public service announcements are contrived and ineffective, and emphasized the importance of developing more prominent, longer, and more realistic campaigns.

“For example, park attendants should check parking and issue fines for those who smoke.” (Participant 7)

“Public service announcements are so contrived, no one takes them seriously. Longer, more attention-grabbing content should be broadcast.” (Participant 10)

“Inspections need to be increased… If there are no fines, no one will follow the rules.” (Participant 10)

## 4 Discussion

The current qualitative study facilitated in-depth discussion of Turkish women’s perceptions, knowledge, beliefs, exposure status and the obstacles they face in preventing exposure to passive smoking in Türkiye. Participants demonstrated some awareness of the potential dangers of SHS exposure but had limited understanding of the specific health problems it can cause. Despite this partial awareness, our study highlights the importance and challenge of creating and maintaining smoke-free environments for women and children in Türkiye, both at home and in public spaces. While formal restrictions are rarely enforced within households, women make efforts to establish rules to protect their families. However, these efforts are often constrained by cultural norms, such as hospitality practices or deference to older family members.

Some participants in this study believed that smoking in designated rooms or near open windows was sufficient to prevent SHS exposure for others, an assumption echoed in previous studies (19–21). This finding indicates that misunderstandings and limited knowledge about SHS may unintentionally lead to ongoing exposure among household members. Such misunderstandings may stem from the lack of comprehensive health education on SHS in Türkiye. Antenatal guidance often focuses on recording family smoking history but seldom explains what SHS is, the harmful substances it contains, or its effects on pregnancy and child development. Supporting this, a cross-sectional survey conducted in Türkiye found that 48 and 89% of pregnant women (*n* = 272) were not asked whether they smoked or were exposed to SHS, respectively (22). This knowledge gap underscores the need for more robust educational efforts to increase awareness. Improving understanding and shifting attitudes toward SHS are crucial, as raising parental awareness of its harms and benefits of protection may effectively encourage the adoption of smoke-free home environments (23).

A case study from Northern India showed that although nursing professionals have strong potential to influence tobacco reduction, existing training and curriculum integration remain insufficient, and further research is needed to strengthen their role in cessation interventions (24). This parallels our findings in Türkiye, where women reported limited SHS-specific counselling during routine care, highlighting a broader global need to better equip frontline health professionals in tobacco control. Similar system-level gaps have been reported in studies involving healthcare professionals themselves. An online survey of doctors in India found high rates of tobacco use and limited awareness about SHS harms and available cessation support services, despite doctors’ willingness to quit (25). The authors noted that even among medical professionals, knowledge of SHS risks and engagement with cessation specialists were low. This parallels our findings in Türkiye, where women reported receiving minimal SHS-specific counselling during routine care, highlighting a broader global need to strengthen tobacco control training and awareness among healthcare providers.

As reported by participants in this study, women’s awareness and efforts to reduce SHS exposure both at home or in public settings intensified during pregnancy and after childbirth, particularly due to their maternal instincts. A study from Türkiye found that the frequency of smoking at home decreased following the birth of a baby, though the reduction was not sufficient to eliminate exposure (26). Similarly, a study from the United Kingdom showed that while the arrival of a newborn did not significantly increase smoking cessation attempts or success among majority of fathers, refraining from smoking inside the home was a more achievable behavioural change. Notably, 78% of fathers had attempted to avoid smoking indoors, and 60% reported doing so successfully (27). These findings highlight the need to engage and support smoking fathers with tailored education and behaviour change interventions during their partner’s pregnancy and the early postpartum period to foster and maintain smoke-free home environments.

Findings from India also reveal that although tobacco research has expanded considerably—especially in biomedical domains such as cancer prevention and tobacco control—social, gendered, and environmental aspects of tobacco exposure remain insufficiently studied (28). Despite methodological advances and increasing publication volume, research on SHS exposure in private spaces, household power dynamics, and women’s vulnerability has received little attention. This mirrors the gaps observed in the Turkish context, where women’s experiences of SHS are shaped not only by health risks but also by cultural norms, domestic hierarchies, and inconsistent enforcement of regulations.

Türkiye has implemented a comprehensive strategy to address tobacco use and its related health risks, aligning closely with the WHO’s MPOWER framework. In 2008, the Law on the Prevention and Control of Hazards of Tobacco Products (Law No. 5727) was amended to address indoor smoking (29). Further efforts were made in 2015, when the Turkish Ministry of Health issued a circular proposing a ban on the use of tobacco and related products in specific outdoor areas of public institutions, as well as in public spaces such as playgrounds, and in areas designated for physical activity, including walking trails and sports fields (30). The enforcement of indoor smoking bans, however, often leads to smoking in nearby outdoor areas, such as patios and smoking in these adjacent outdoor spaces appears to increase SHS exposure in both outdoor and indoor environments (31). Therefore, the current indoor SHS legislation should be extended to cover adjacent outdoor areas of venues in order to effectively protect people from SHS in Türkiye.

### 4.1 Strengths and limitations

To our knowledge, the current qualitative study is the first qualitative study that explored Turkish women’s perceptions, knowledge, beliefs, exposure status and the obstacles they face in preventing exposure to passive smoking. Participants were recruited from multiple cities across Türkiye; however, all held at least a bachelor’s degree. This means that perspectives of women with lower educational backgrounds who may experience different forms or levels of SHS exposure and have varying degrees of health literacy are not fully represented. Therefore, the transferability of findings to less-educated or illiterate populations may be limited and should be approached with caution.

### 4.2 Implications for practice and future research

The findings of this study indicate that SHS, while often overlooked, remains a significant problem for women. Therefore, individual education should not be limited to women; regulations encompassing the family, community, and public spaces should be implemented to protect women from SHS. While legal regulations restrict smoking in public spaces, implementation appears inadequate, and SHS, particularly at home, is not fully addressed. Therefore, it is vital that policymakers strengthen sanctions, expand regulations to include smoke-free areas, and develop awareness campaigns tailored to family habits. Furthermore, necessary sanctions and controls regarding SHS should be increased in public spaces and many other areas. Furthermore, new products such as e-cigarettes remain available in informal markets despite partial sales restrictions. These products introduce new pathways of aerosol exposure, highlighting the need for stronger regulations and public education for young people.

In line with the WHO MPOWER framework (32) and Türkiye’s current tobacco control laws, several actionable steps can be taken. Enforcement of smoke-free regulations should be strengthened in parks, playgrounds, outdoor cafés, and semi-open areas, with municipal officers conducting regular inspections and issuing fines. Healthcare professionals should integrate SHS screening into routine maternal and child health visits and provide brief counselling to both smokers and non-smokers. School-based SHS awareness programmes developed with the Ministry of National Education could further support prevention efforts. Additionally, national media campaigns should use more engaging and realistic content, and building regulations should be updated to reduce smoke drift between apartments through improved ventilation and smoke-free common areas.

The findings suggest that healthcare professionals (especially nurses and doctors) should more effectively raise the issue of SHS during routine checkups. Counselling should be provided not only to smokers but also to their non-smoking family members, providing them with the knowledge and skills necessary to advocate for a smoke-free environment. Finally, future research could include longitudinal studies to explore the health impacts of long-term exposure to SHS in women and children in Türkiye. Furthermore, similar qualitative studies could be conducted on specific groups, such as youth, pregnant women, or healthcare workers, and compared with the literature.

Overall, this study demonstrates that reducing SHS exposure requires a multifaceted approach. More lasting results can be achieved when individual empowerment, family communication, public awareness, and effective policy implementation are considered together.

## Data Availability

A data set will be available upon request. Requests to access the datasets should be directed to hatice.phd.sheffield@gmail.com.

## References

[1] World Health Organization. Warning about the dangers of tobacco. WHO report on the global tobacco epidemic (2025). Available online at: https://www.who.int/publications/i/item/9789240112063 (Accessed July 7, 2025)

[2] Royal College of Physicians of London. Passive smoking and children: a report. A report by the tobacco advisory group. London: RCP (2010). Available online at: https://shop.rcp.ac.uk/products/passive-smoking-and-children?variant=6634905477 (Accessed July 11, 2025)

[3] Hassanein ZM Nalbant G Bogdanovica I Langley T Murray RL. A qualitative study of barriers and motivators to prevent secondhand smoke exposure among pregnant women and children in Egypt: identifying appropriate approaches for change. Nicotine Tob Res. (2024) 26:1545–52. doi: 10.1093/ntr/ntae051 38780225

[4] Pugmire J Sweeting H Moore L. Environmental tobacco smoke exposure among infants, children and young people: now is no time to relax. Arch Dis Child. (2017) 102:117–8. doi: 10.1136/archdischild-2016-311652 28100555

[5] U.S. Department of Health and Human Services. The health consequences of smoking—50 years of progress: a report of the surgeon general. Atlanta, GA (2014). U.S. Department of Health and Human Services (Centers for Disease Control and Prevention). Available online at: https://www.hhs.gov/sites/default/files/consequences-smoking-exec-summary.pdf

[6] Murray RL Britton J Leonardi-Bee J. Second hand smoke exposure and the risk of invasive meningococcal disease in children: systematic review and meta-analysis. BMC Public Health. (2012) 12:106–15. doi: 10.1186/1471-2458-12-1062 23228219 PMC3534009

[7] Jones LL Hashim A Mckeever T Cook DG Britton J Leonardi-bee J. Parental and household smoking and the increased risk of bronchitis, bronchiolitis and other lower respiratory infections in infancy: systematic review and meta-analysis. Respir Res. (2011) 12:1–11. doi: 10.1186/1465-9921-12-5 21219618 PMC3022703

[8] Lee KA Palipudi KM English LM Ramanandraibe N Asma S. Secondhand smoke exposure and susceptibility to initiating cigarette smoking among never-smoking students in selected African countries: findings from the global youth tobacco survey. Prev Med (Baltim) (2016), 91S: S2–S8. Available online at: 10.1016/j.ypmed.2016.04.017, 27138692

[9] Tan CE Glantz SA. Association between smoke-free legislation and hospitalizations for cardiac, cerebrovascular, and respiratory diseases: a meta-analysis. Circulation. (2012) 126:2177–83. doi: 10.1161/CIRCULATIONAHA.112.121301, 23109514 PMC3501404

[10] Frazer K Je C Mchugh J S VB Clarke A Doherty K . Legislative smoking bans for reducing harms from secondhand smoke exposure, smoking prevalence and tobacco consumption. Cochrane Database Syst Rev. (2016) 2. doi: 10.1002/14651858.CD005992.pub3 PMC6486282 26842828

[11] Been JV Mackay DF Millett C Pell JP Van SOCP Sheikh A. Impact of smoke-free legislation on perinatal and infant mortality: a national quasi-experimental study. Sci Rep. (2015) 5:13020. doi: 10.1038/srep13020 26268789 PMC4534797

[12] Jarvis MJ Mindell J Gilmore A Feyerabend C West R. Smoke-free homes in England: prevalence, trends and validation by cotinine in children. Tob Control. (2009) 18:491–5. doi: 10.1136/tc.2009.031328, 19748885

[13] Hassanein ZM Nalbant G Langley T Murray RL Bogdanovica I Leonardi-Bee J. Experiences and views of parents on the prevention of second-hand smoke exposure in middle eastern countries: a qualitative systematic review. JBI Evid Synth. (2022) 20:1969–2000. doi: 10.11124/jbies-21-00222, 35971199

[14] Elbek O Kılınç O Salepçi B Bostan P Çetinkaya PD Arpaz S . Tobacco control in Turkey in the light of the global adult tobacco survey. Turk Thorac J. (2021) 22:90–2. doi: 10.5152/TurkThoracJ.2021.19139 33646111 PMC7919432

[15] Turkish Ministry of Health. Health statistics yearbook 2021. Ankara: Ministry of Health (2021). Available online at: https://dosyasb.saglik.gov.tr/Eklenti/45317/0/siy2021-ingilizcepdf.pdf (Accessed November 11, 2025)

[16] Kılınç A Çam A Önsüz M Metintaş S. Prevalence of second-hand smoke exposure in houses in Turkey: a meta-analysis study. Eur J Pub Health. (2020) 30:ckaa166–142. doi: 10.1093/eurpub/ckaa166.142

[17] Tong A Sainsbury P Craig J. Consolidated criteria for reporting qualitative research (COREQ): a 32-item checklist for interviews and focus groups. Int J Qual Health Care. (2007) 19:349–57. doi: 10.1093/intqhc/mzm042, 17872937

[18] Braun V Clarke V. Using thematic analysis in psychology. Qual Res Psychol. (2006) 3:77–101. doi: 10.1191/1478088706qp063oa

[19] Abdul Mutalib RNS Abd Rani NL Zulkifli A Abd Latif NH Dobson R Engku Ibrahim TA . Knowledge, beliefs, and behaviors related to secondhand smoke and smoking in the home: a qualitative study with men in Malaysia. Nicotine Tob Res. (2023) 25:821–7. doi: 10.1093/ntr/ntac239 36239239 PMC10032199

[20] Jones LL Atkinson O Longman J Coleman T McNeill A Lewis SA. The motivators and barriers to a smoke-free home among disadvantaged caregivers: identifying the positive levers for change. Nicotine Tob Res. (2011) 13:479–86. doi: 10.1093/ntr/ntr030, 21447837

[21] Rosen LJ Lev E Guttman N Tillinger E Rosenblat S Zucker DM . Parental perceptions and misconceptions of child tobacco smoke exposure. Nicotine Tob Res. (2018) 20:1369–77. doi: 10.1093/ntr/ntx169, 29059387

[22] Cengizoğlu H Gölbaşı Z. Gebe Kadinlarin Si̇gara Kullanimi ve Pasi̇f Si̇gara Dumanı Maruzi̇yeti̇ni̇n Beli̇rlenmesi̇. Gazi Sağlık Bilim Derg. (2021) 6:78–89. doi: 10.52881/gsbdergi.938147

[23] Chansaeng S Boonchieng W Naksen W. Secondhand smoke prevention through the perceptions of pregnant women with smoking family members: a Thailand study. Int J Qual Stud Health Well-being. (2024) 19:2326109. doi: 10.1080/17482631.2024.2326109, 38498815 PMC10949832

[24] Gupta R Goel S Bashar A Bhattacharya S Singh R. Level of teaching of tobacco control measures and cessation interventions in a nursing care institute of northern India- a case study. J Community Health Manag. (2017) 4:35–7. doi: 10.18231/2394-2738.2017.0007

[25] Gupta P Garg S Singh R Bhattacharya S. An online survey of doctor’s willingness to quit tobacco in India. J Family Med Prim Care. (2025) 14:2313–7. doi: 10.4103/jfmpc.jfmpc_1096_24, 40726696 PMC12296327

[26] Aslan D Daymaz D Gürsoy N Kartal G Yavuz M. Status of exposure to second-hand smoke at home in children under five years of age: an example from Ankara province. Turk Thorac J. (2015) 16:16–21. doi: 10.5152/ttd.2014.4180, 29404072 PMC5783041

[27] Blackburn C Bonas S Spencer N Dolan A Coe C Moy R. Smoking behaviour change among fathers of new infants. Soc Sci Med. (2005) 61:517–26. doi: 10.1016/j.socscimed.2004.12.009, 15899312

[28] Singh A Kashyap A Varshney S Bhattacharya S. Decades of discovery: unveiling emerging trends, pivotal research areas, and landmark publications in national tobacco research in India. Front Res Metr Anal. (2025) 10:1496571. doi: 10.3389/frma.2025.1496571, 40678731 PMC12267211

[29] Official Gazette of the Republic of Turkey. Law on the amendment of the law on the prevention of hazards to tobacco products (2008). Available online at: https://assets.tobaccocontrollaws.org/uploads/legislation/Turkey/Turkey-Law-No.-4207.pdf (Accessed August 10, 2025).

[30] National Public Health Agency of Turkey. Tobacco control strategic document and action plan 2018–2023 (2018). Available online at: https://havanikoru.saglik.gov.tr/havanikoru/dosya/eylem_plani_ve_strateji_tutun_HD.pdf (Accessed July 25, 2025)

[31] Sureda X Fernández E López MJ Nebot M. Secondhand tobacco smoke exposure in open and semi-open settings: a systematic review. Environ Health Perspect. (2013) 121:766–73. doi: 10.1289/ehp.1205806, 23651671 PMC3701994

[32] World Health Organization. WHO report on the global tobacco epidemic, 2008: The MPOWER package. Geneva: World Health Organization (2008). Available online at: https://www.who.int/publications/i/item/9789241596282 (Accessed November 11, 2025)

