# Supplementary material for: Perceptions, knowledge and beliefs of women regarding second-hand smoke exposure: a qualitative study to identify approaches for change
Source: Front Public Health. 2026 Feb 5;14:1716775. doi: 10.3389/fpubh.2026.1716775 (PMC12918748; doi:10.3389/fpubh.2026.1716775)
Supplement: Supplementary file 1 [file Table_1.docx]

**Date:**

**Dear Participant,**

This study has been designed to examine Turkish women’s perceptions, knowledge, beliefs, exposures, and existing barriers regarding second-hand smoke. Second-hand smoke, also known as passive smoking, refers to inhaling tobacco smoke from others.

You have the right to refuse participation or to withdraw from the study at any time without providing a reason. This session will be audio-recorded and transcribed, and anonymized quotations may be used in future reports or publications. To explore your experiences with second-hand smoke, I will ask you some questions; there are no right or wrong answers — our aim is to understand your views.

If you decide to withdraw from the study on your own or if the researcher excludes you from the scope of the study, no penalty will be applied and there will be no disadvantage to you. You will not be asked for any identifying information. Participation in the study is entirely voluntary, and no payment will be requested or provided.

All information obtained during the research will be kept strictly confidential, and your name will not appear in any report or publication. Your answers will be kept completely confidential and evaluated only by the researchers; the information obtained will be used in scientific publications.

Your responses are important for the reliability of the research, so we kindly ask you to answer accurately and completely. If you would like more information about the project, you can contact the research team.

**Thank you very much for your participation.**

**Research Team**
Assist. Prof. Hatice Bulut
haticebulut@sdu.edu.tr

Assist. Prof. Gamze Nalbant
[gamzeustundag93@gmail.com](mailto:gamzeustundag93@gmail.com)

Dr. Zeinab M. Hassanein

# Appendix 1. Sociodemographic Data Form

1. Age (in full years): ..........................................

2. Place of residence: (1. Rural 2. Urban)

3. City of residence: ..........................................

4. Marital status: (1. Single 2. In a relationship 3. Married 4. Widowed 5. Divorced)

5. Do you have children? (1. Yes 2. No)

6. Number of children: ..........................................

7. Age(s) of child/children: .............................

8. Are you currently pregnant? (1. Yes 2. No)

9. Religion: ..........................................

10. Highest level of education: (1. Primary school 2. Secondary school 3. High school 4. Associate degree 5. Bachelor’s degree 6. Postgraduate degree)

11. Are you employed? (1. Yes 2. No)

12. Occupation: ..........................................

13. Economic status: (1. Income more than expenses 2. Income equal to expenses 3. Income less than expenses)

14. Do you smoke? (1. Yes 2. No)

15. Are you exposed to second-hand smoke? (1. Yes 2. No)

16. Spouse’s age: ..........................................

17. Spouse’s education level: (1. Primary school 2. Secondary school 3. High school 4. Associate degree 5. Bachelor’s degree 6. Postgraduate degree)

18. Does your spouse smoke? (1. Yes 2. No)

19. Do any family members smoke? ..........................................

Other individuals living in the household: ..........................................

20. Chronic illness: (1. Yes, specify 2. No, I do not have a chronic illness)

21. Do you regularly use any medication? (1. Yes 2. No)

## Knowledge and Attitudes Regarding Second-Hand Smoke

1. What do you think about the possible harms of second-hand smoke, especially in terms of general health and women’s health?
   •
2. How do you think exposure to second-hand smoke affects the health of pregnant women? What might this exposure lead to? What harms could it cause for both the mother and the baby?
   •
   Have you ever encountered a woman or a baby who experienced health problems due to this reason?
   •

**NOTE:** Compared to active smoking, exposure to second-hand smoke carries similar risks for pregnant women. Women exposed to second-hand smoke are more likely to give birth to babies with lower birth weight, shorter birth length, smaller head circumference, and stillbirth. For non-smokers, there is no safe level of exposure to second-hand smoke.

1. How do you think second-hand smoke affects children’s health? Do you think it differs depending on the child’s age?
   •
2. Where are you exposed to second-hand smoke? (home, work, park, social spaces, etc.)
   •
3. Who smokes in your home or workplace? Do they regularly smoke near you?
   •
4. What do you think about being exposed to second-hand smoke? Why?
   •
5. What do you think about changing your exposure to second-hand smoke? Why?
   •
6. Where do you think you could change this exposure?
   •
7. Has anything ever changed your beliefs about second-hand smoke? What could change your opinion? (Personal experience, society, etc.)
   •
8. How do you think exposure to second-hand smoke among pregnant women and children is perceived by society? Why? (Are there any societal recommendations about not smoking near pregnant women or children?)
   •

These were the questions related to your knowledge about second-hand smoke exposure. Would you like to add anything else?
•

## Household Smoking Rules

1. Do you have any household rules about smoking? What are they? Do you have restrictions on smoking in the presence of children? (Are there special rules for children? Did you change these rules depending on the child’s age? At what age did you make this change?)
   •
2. For any family, why might there be a smoking ban at home?
   •
3. During your pregnancy, could you provide more details about smoking at home? (Were there any changes in behavior or frequency? Did your spouse, for example, try to quit smoking? How? What motivated him?)
   •
4. Have you taken any measures to reduce your children’s exposure to second-hand smoke? What are they? (For example, persuading your husband to smoke outside, away from the children.)
   •
5. What prevents you from adopting a smoke-free home? For those who tried, did it work and what challenges did you face? For those who succeeded, what helped?
   •
6. Is there anything that prevents you from protecting pregnant women and children at home from exposure to second-hand smoke? Work-related reasons?
   •
7. What measures could help prevent exposure of pregnant women and children to second-hand smoke at home? Who should implement these measures? These were all the questions related to household smoking rules. Would you like to add anything else?
   •

## Counseling Services on Reducing Second-Hand Smoke

1. Where did you receive information about the health risks of second-hand smoke? (family doctor, nurse, friends, family, TV/media/advertisements, social media such as Instagram, Facebook, X, etc.)
   •
2. Has your primary healthcare doctor/nurse ever asked you about exposure to second-hand smoke? What did they discuss with you? (What information did you receive from them?)
   •
3. Did you make any changes as a result of this information? Was it different depending on whether you received the information from TV/media advertisements or from primary healthcare providers?
   •
4. Health education interventions are designed to provide individuals with health information to improve their health and well-being (such as the Ministry of Health’s educational interventions on the importance of breastfeeding). Therefore, what do you think about offering an educational intervention in primary healthcare centers to reduce second-hand smoke exposure among pregnant women and children? What do you think would be the best method? (Face-to-face? Printed educational materials? Public lectures? Health education sessions for non-smoking mothers? Including husbands in counseling sessions? Providing husbands with nicotine replacement aids such as patches—do you think husbands would accept counseling and patches?)
   •
5. If the Ministry of Health implemented a smoke-free home intervention, would you participate? What would motivate you? What challenges would you face?
   •
6. Considering the smokers in your household, would they be willing to participate in such educational interventions?
   •
7. Do you think nurses, especially women’s health and family health center nurses, should inform women about this issue?
   •

## Final Question

1. Is there anything else that you think might be useful, but that we have not discussed? Would you like to add anything? Or would you like us to use this for improving future discussion groups?
   •
